# Tumor Cells Switch to Mitochondrial Oxidative Phosphorylation under Radiation via mTOR-Mediated Hexokinase II Inhibition - A Warburg-Reversing Effect

**DOI:** 10.1371/journal.pone.0121046

**Published:** 2015-03-25

**Authors:** Chung-Ling Lu, Lili Qin, Hsin-Chen Liu, Demet Candas, Ming Fan, Jian Jian Li

**Affiliations:** Department of Radiation Oncology, University of California Davis School of Medicine, Sacramento, California, United States of America; University of Pittsburgh School of Medicine, UNITED STATES

## Abstract

A unique feature of cancer cells is to convert glucose into lactate to produce cellular energy, even under the presence of oxygen. Called aerobic glycolysis [The Warburg Effect] it has been extensively studied and the concept of aerobic glycolysis in tumor cells is generally accepted. However, it is not clear if aerobic glycolysis in tumor cells is fixed, or can be reversed, especially under therapeutic stress conditions. Here, we report that mTOR, a critical regulator in cell proliferation, can be relocated to mitochondria, and as a result, enhances oxidative phosphorylation and reduces glycolysis. Three tumor cell lines (breast cancer MCF-7, colon cancer HCT116 and glioblastoma U87) showed a quick relocation of mTOR to mitochondria after irradiation with a single dose 5 Gy, which was companied with decreased lactate production, increased mitochondrial ATP generation and oxygen consumption. Inhibition of mTOR by rapamycin blocked radiation-induced mTOR mitochondrial relocation and the shift of glycolysis to mitochondrial respiration, and reduced the clonogenic survival. In irradiated cells, mTOR formed a complex with Hexokinase II [HK II], a key mitochondrial protein in regulation of glycolysis, causing reduced HK II enzymatic activity. These results support a novel mechanism by which tumor cells can quickly adapt to genotoxic conditions via mTOR-mediated reprogramming of bioenergetics from predominantly aerobic glycolysis to mitochondrial oxidative phosphorylation. Such a “waking-up” pathway for mitochondrial bioenergetics demonstrates a flexible feature in the energy metabolism of cancer cells, and may be required for additional cellular energy consumption for damage repair and survival. Thus, the reversible cellular energy metabolisms should be considered in blocking tumor metabolism and may be targeted to sensitize them in anti-cancer therapy.

## Introduction

Two different bioenergetics pathways are utilized in mammalian cells dependent on oxygen status. When cells have sufficient oxygen, they will metabolize one molecule of glucose into approximately 34 molecules of ATP via oxidative phosphorylation (OXPHOS) in the mitochondria, producing the major cellular fuels for energy consumption. In contrast, under hypoxic conditions, cells metabolize one molecule of glucose into two molecules of lactate and this energy metabolism can only create two molecules of ATP [1]. In 1956, Otto Warburg discovered that cancer cells tend to convert glucose into lactate to produce energy rather than utilizing OXPHOS, even under oxygenated conditions. This phenomenon is called aerobic glycolysis, also known as the Warburg effect [2, 3]. It is believed that tumor cells metabolize glucose to lactate to use the intermediates of glycolysis to support cell proliferation at the expense of producing less energy [1]. However, recent studies indicate that the increase of aerobic glycolysis does not fully replace the mitochondrial functions in cancer cells; they still can increase respiratory activity [4–8]. Importantly, it is known that reoxygenation in hypoxic tumors during radiation treatment causes a shift from an hypoxic environment to a more oxygenated condition, due to death of tumor cells and the reconstruction of vasculature [9]. It remains unclear whether aerobic glycolysis in tumor cells is reversible, back to oxidative phosphorylation, under specific genotoxic stress conditions such as ionizing radiation (IR) exposure. Here we report that mTOR, highly expressed in many human tumors [10], plays a key role in switching aerobic glycolysis back to oxidative phosphorylation. This demonstrates a unique mechanism by which cancer cells can generate increased cellular energy, potentially useful as an aid to enhance cellular survival under therapeutic genotoxic stress conditions.

Mammalian target of rapamycin (mTOR) is a Serine/Threonine kinase that belongs to the PI3K family. It can regulate an array of cellular functions including protein synthesis, metabolism, and cell proliferation. mTOR has been shown to form two distinct complexes with different functions [11]: mTOR complex 1 (mTORC1) and mTOR complex 2 (mTORC2). mTORC1 is composed of mTOR, raptor, PRAS40 and mLST8/GbL. It has two well-defined substrates: p70 ribosomal S6 kinase 1 (referred to S6K1) and 4E-BP1, both can regulate protein synthesis [12]. The critical functions of mTORC1 include DNA double-strand break repair [13] and mitochondria function [14–19], and mTORC1 itself can play a negative feedback role in the PI3K/Akt/mTOR pathway via activation of its substrate, S6K1 that controls the signal influx by inhibiting the receptors when it is activated [20–23]. Therefore, the feedback system can down-regulate protein synthesis to control cell proliferation. The second complex, mTORC2, is composed of mTOR, rictor, mSIN1, protor, and mLST8 able to phosphorylate Akt, an upstream regulator of mTORC1, to control the signaling pathway [24], suggesting a across-talk between mTORC1 and mTORC2. mTORC2 has also been indicated in the control of cellular metabolism in glioblastoma via c-Myc regulation [25].

Mutations in the mTOR signaling pathway have been found in breast and renal cancer [22, 24] and thus mTOR has been targeted in several clinical trials [26, 27]. Most of the studies focused on the inhibition of mTOR by using mTOR specific inhibitors, rapamycin and its analogs. However, although these inhibitors are shown to be able to block mTOR activity and inactivate its down-stream substrates to reduce protein synthesis [20], inhibition of mTOR is only significant *in vitro*, the clinical test for these inhibitors did not show significant treatment improvement [28, 29]. Currently, it is unknown how these tumor cells are resistant to anti-mTOR treatments. Since mTOR is related to cell energy metabolism, any inhibition or mutation on mTOR can change the way cells produce their energy. It has been well-documented that mTOR dysfunction can transform normal cells into tumor-like cells [30] and switch the energy metabolism from mitochondria-centered oxidative phosphorylation to aerobic glycolysis [31]. However, a critical question is whether a transformed cell or tumor cell can “re-use” mitochondrial oxidative phosphorylation to generate the necessary cellular bioenergetics for survival if aerobic glycolysis is blocked. Interestingly, mTOR has been detected on the mitochondria surface [17], and inhibition of mTOR can decrease the function of mitochondrial metabolism such as oxygen consumption, mitochondrial membrane potential and the TCA cycle [14, 15]. Here, we provide evidence indicating that under radiation stress, tumor cells can relocate mTOR to mitochondria where it interacts with Hexokinase II, the enzyme that phosphorylates glucose in glycolysis and an inhibitor factor to mitochondrial metabolism [32, 33], leading to an enhanced mitochondrial oxidative phosphorylation with increased mitochondrial ATP generation. The switch of bioenergetics from aerobic glycolysis to mitochondrial oxidative phosphorylation is shown to increase tumor resistance to radiation treatment. Thus, blocking the mitochondrial respiration together with the inhibition of aerobic glycolysis may significantly enhance the effectiveness of current anti-cancer therapy.

## Materials and Methods

### Cell culture and reagents

The human breast epithelial cell line, MCF-10A, was maintained in DMEM medium supplemented with 10% horse serum (HyClone, Logan, UT), penicillin (100 units per ml) and streptomycin (100 μg/ml), 20 ng/ml epidermal growth factor, 100 ng/ml cholera toxin, 0.5 μg/ml hydrocortisone and 10 μg/ml insulin; the mouse breast epithelial cancer cell line, 4T1, was maintained in DMEM medium supplemented with 10% fetal bovine serum, 100 unit/ml penicillin and 100 μg/ml streptomycin; human prostate epithelial *cell* line, 267B1, and K-Ras transformed prostate epithelial cell line, 267B1/Ki, were maintained in DMEM medium supplemented with 5% fetal bovine serum, 100 unit/ml penicillin and 100 μg/ml streptomycin; the human breast epithelial cancer cell line, MCF-7, and glioblastoma cell line, U87, were maintained in MEM medium supplemented with 10% fetal bovine serum, 100 unit/ml penicillin and 100 μg/ml streptomycin, 0.1 mM non-essential amino acids and 1 mM sodium pyruvate; the human colon cancer cell line, HCT116, was maintained in McCoy`s 5A medium supplemented with 10% fetal bovine serum, 100 unit/ml penicillin and 100 μg/ml streptomycin. All cell lines were maintained in a humidified incubator at 37°C (5% CO_2_). All cell lines were originally purchased from ATCC. Rapamycin (SC-3504) was purchased from Santa Cruz Biotechnology Inc., (Santa Cruz, CA, USA).

### Measurement of mitochondrial ATP production

Mitochondrial ATP generation was measured following the described protocol [34]. Briefly, cells were seeded in 96-well plates and treated with 5 Gy of radiation. After treatment, cells were collected and washed with cold PBS twice, before they were incubated in 25 μg/ml digitonin for 1 min on ice, digitonin removed and cells washed twice with cold PBS. 5% TCA was then added to cells with P1,P5-di (adenosine) pentaphosphate (to 0.15 mM), ADP (to 0.1 mM), malate plus pyruvate (both to 1 mM), one replicate for each sample was prepared containing the components described above plus 1 mg/ml oligomycin, in a total volume of 40 μl. Plates were shaken 20 min on ice to extract ATP, followed by the addition of 140 μl of 250 mM Tris-Acetate (pH 7.75) in each well. The ATP extracts were used to measure ATP production using the ATP determination kit [Invitrogen] according to the manufacturer’s instructions, using a luminometer for readout (Turner Biosystem, Sunnyvale, CA). A standard curve was plotted with known ATP concentrations ranging from 100 pM to 1 mM. The ATP production from control groups was set as 100%.

### Lactate assay

Lactate production was measured using BioVision lactate colorimetric assay kit II (#K627–100). Cells were cultured in 10 cm plates and collected in the lactate assay buffer provided by the assay kit, homogenized and then changes in lactate production measured using a SpectraMax M2 MultiMode Microplate Reader (Molecular Devices, Sunnyvale, CA, USA), with OD 450 nm.

### Oxygen consumption assay

Oxygen consumption was measured following the established method [35] with some modification. Cells were seeded in 10 cm plates, treated with 5 Gy of radiation and collected after 24 h. Cell pellets were re-suspended in 300 μl of respiratory buffer (250 mM sucrose, 15 mM KCl, 1 mM EGTA, 5 mM MgCl_2_ and 30 mM K_2_HPO_4_, pH7.4), 25 mM Succinate and 25 μg/ml Digitonin. Resuspended cells were transferred to the electrode on dual digital model 20 oxygen measurement controllers (Rank Brothers Ltd). After 500 seconds of measurement, 1.65 mM ADP was added to trigger the respiratory chain reaction, then 2.5 μg/ml oligomycin added to stop the reaction. The total measurement was stopped at 500 seconds. The oxygen consumption rate was calculated in nmol/min.

### Isolation of mitochondrial fraction

Mitochondrial fractions were extracted from exponentially growing cells at 50–80% confluence using a mitochondria isolation kit (Thermo Scientific). Briefly, cells were incubated in ice-cold hypotonic buffer containing 10 mM NaCl, 1.5 mM MgCl_2_ and 10 mM Tris—HCl, pH 7.5 for 20 min and the cell membranes disrupted by glass pestle in buffer containing 2 M sucrose, 35 mM EDTA and 50 mM Tris—HCl, pH 7.5. The mitochondrial fractions were then separated by centrifugation at 10000 g for 20 min.

### Western blotting

Immunoblotting was performed as described [36]. Cellular extracts were fractionated using a 5% SDS-polyacrylamide gel and transferred to polyvinylidene difluoride membranes (Bio-Rad) using Bio-Rad semi-dry machine. Immunoblot analysis was visualized using the secondary antibody conjugated with horseradish peroxidase followed by the ECL Western blotting detection system (Amersham Biosciences). Anti-mTOR (2972s), COXIV (4844s) were purchased from Cell Signaling Technology Inc. (Denver, MA, USA). Anti-β-Actin (A5441) was purchased from Sigma-Aldrich (St. Louis, MO, USA). Anti-Hexokinase II (AB3279) was purchased from Millipore (Billerica, MA). Anti-TOM 40 (sc-365467) was purchased from Santa Cruz Biotechnology Inc. (Santa Cruz, CA, USA).

### DNA content flow cytometry

Cultured MCF-7 cells were treated with 5 Gy of radiation and collected at different time points. 1×10^6^ cells were placed in 5 ml PBS and centrifuged at 1500 rpm for 5 minutes. Cells were resuspended thoroughly in 0.5 ml PBS to achieve a single cell suspension then fixed with 4.5 ml 70% ethanol for more than 2 h. Cells were centrifuged at 1500 rpm for 5 min to decant ethanol and washed with 5 ml PBS. Cell pellet was suspended in 1 ml propidium iodide staining solution for 15 min at 37°C before performing flow cytometry.

### 4T1 mouse breast cancer model irradiated in vivo

Immune-competent (Balb/c3) female mice were injected with 1x10^6^ mouse breast cancer 4T1 cells into the mammary glands of both sides. Local tumor radiation treatment was delivered to one tumor when the tumor sizes reached ~5 mm in diameter with a single 5 Gy dose delivered locally to the tumor using an Elekta Beam Modulator Agility linear accelerator (Elekta AB, Crawley, UK) and the tumor of the other side was shielded and used as the non-radiation control. The dose was delivered with 6 MeV electrons directed though a 1 cm cut out for tumor coverage and maximal sparing of the surrounding tissues. A bolus of 0.5 cm "superflab" material was used over the tumor site, which ensured full dose coverage up to the skin surface and further spared the underlying tissues. The thermoluminescent detectors (TLD) were used to calibrate the small field dose with an accuracy of ± 2%. The accuracy of dose delivery was confirmed with a 3D printed phantom representation of the mouse with tumors on each flank. The dose to the irradiated target was measured within 1% of the prescription with a MOSFET detector, with the sham irradiated side measured at only 4% of the prescription dose. Mice were euthanized in CO_2_ chamber 24 h post-irradiation and tumor tissues were extracted for the measurement of mitochondrial oxygen consumption, ATP production, lactate production and western blotting.

### Ethical statements

The animal study was carried out in strict accordance with the recommendations in the Guide for the Care and Use of Laboratory Animals of the National Institutes of Health. The protocol was approved by the Committee on the Ethics of Animal Experiments of the University of California Davis (Permit Number: 15315). All studies involving animals are reported in accordance with the ARRIVE guidelines [37].

### Immunocytochemistry analysis

Cultured MCF-7 cells were seeded on round coverslips and grown to near-confluency. The cells were washed with PBS, fixed in 4% paraformaldehyde (pH 7.2) for 15 minutes and permeabilized with 0.1% Triton X-100 in PBS for 5 minutes. The cells were then incubated in blocking solution for 15 minutes before the primary antibody incubation overnight at 4°C with 1:200 dilutions. Cells were incubated with TR- or FITC-conjugated secondary antibodies diluted 1:1000 in the blocking solution for 1 h at room temperature in the dark before analysis with confocal microscopy.

### Clonogenic survival assay

Cells were seeded in 60 mm dishes with or without 5 Gy or rapamycin treatments. The treated and control cells were cultured for 14 days and colonies with more than 50 cells were scored and normalized to the plating efficiency of each cell line.

### Co-Immunoprecipitation (Co-IP)

Co-IP was conducted with MCF-7 cells 24 h after 5 Gy irradiation as previously described [38] with anti-mTOR antibody. Precipitants were further subjected to western blotting using anti-Hexokinase II (AB3279; Millipore, Billerica, MA). Normal IgG was used as a negative control and the whole cell lysate without IP was included as positive control.

### Hexokinase activity assay

MCF-7 cells were collected at sham and 24 h post-5 Gy radiation with or without rapamycin. Cells were lysed and the activity measured following the Hexokinase Assay Kit protocol (Cat# E-111) from Biomedical Research Service Center using a SpectraMax M2 MultiMode Microplate Reader (Molecular Devices, Sunnyvale, CA, USA), with OD 492 nm. The activity was calculated in IU/L unit.

### Statistical analysis

Data are presented as mean ± SEM and statistical analyses were performed using paired student t-test.

## Results

### The location of mTOR is related to cellular bioenergetics

**Fig 1:**
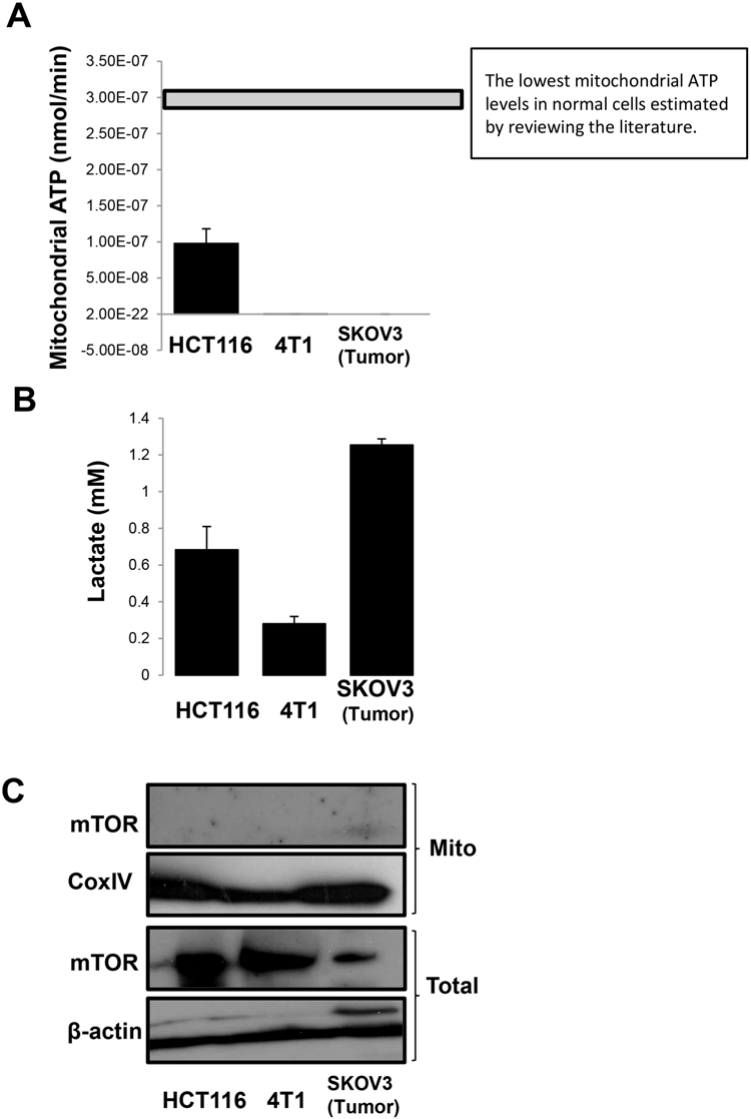
Aerobic glycolysis [Warburg effect] was detected in human, mouse and xenograft tumors without mitochondrial mTOR localization. (A) Cellular mitochondrial ATP production and (B) lactate production and (C) mTOR western blotting in total cell lysates and mitochondrial fractions in HCT116 cells, 4T1 cells and SKOV3 xenograft.

**Fig 2:**
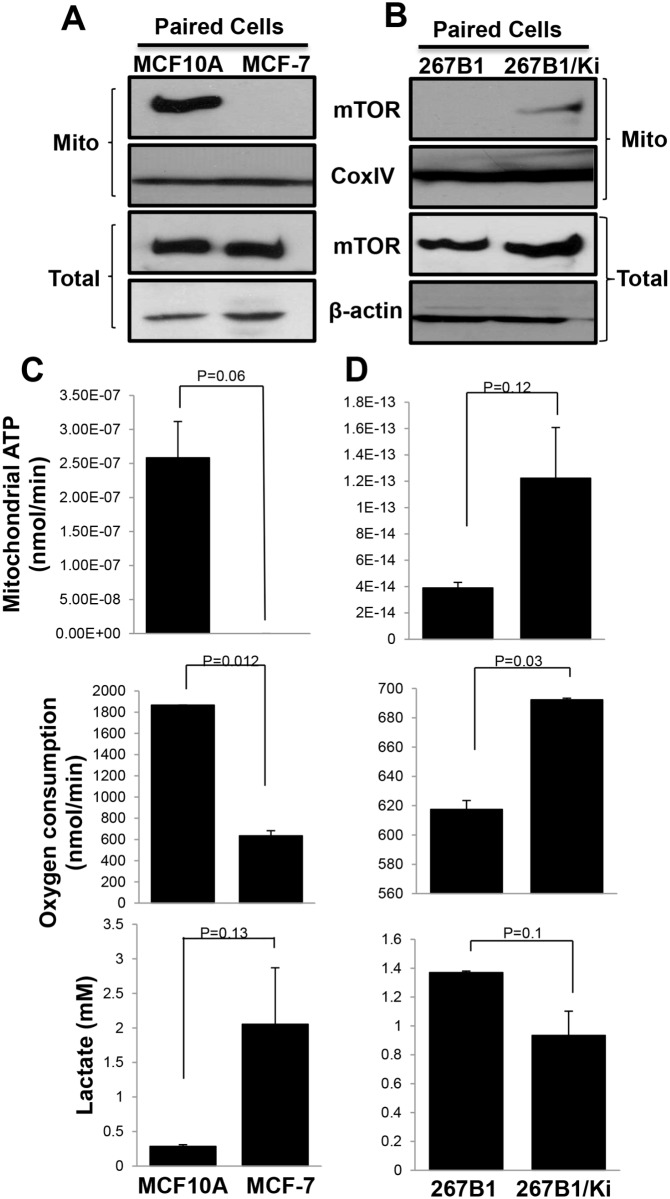
Mitochondrial mTOR contributed to mitochondrial respiration. mTOR western blotting was performed using total cell lysates or mitochondrial fractions in paired normal and breast cancer MCF-10A, MCF-7 cells (A) and paired normal and transformed prostate cancer 267B1, 267B1/Ki cells (B). Cellular mitochondrial ATP production, oxygen consumption and lactate production of (C) MCF-10A and MCF-7, (D) 267B1 and 267B1/Ki cell lines were measured to compare the bioenergetics difference in two pairs of cells. Data are mean ± SEM, p values were included in each data.

As reported by others, aerobic glycolysis observed in many cancer cells may be tracked by its reduced ATP generation and concomitant increase in lactate output. It is thus a potentially useful marker for transformed or tumor cells. Consistent with the features of aerobic glycolysis, the Warburg effect, lowered mitochondrial ATP generation (Fig. 1A) and enhanced glycolysis using lactate production as the indicator (Fig. 1B), were detected in human colon cancer HCT116, murine breast cancer 4T1 cells and human ovarian SKOV3 xenograft tumor tissue compared with the levels reported in normal cells [39–42]. mTOR is linked to the proliferation of cancer cells and can control the transcription of key enzymes that promote aerobic glycolysis [24]. Inhibition of mTOR is shown to impair the functions of mitochondria and increase lactate production in Jurkat cells [14, 22]. We hypothesized that mTOR may have a function in mitochondrial metabolism. However, there was no detectable mTOR proteins in the mitochondrial fractions prepared from the tumor cells and tissues (Fig. 1C). Then, we further examined mTOR mitochondrial location and cellular bioenergetics in paired normal and cancer cells, including normal human breast epithelial cells MCF-10A and breast cancer MCF-7 cells; and human prostate epithelial cells 267B1 and their K-Ras transfected derivative, 267B1/Ki (Figs. 2A and 2B). Interestingly, the mitochondrial ATP generation was reduced with lowered oxygen consumption in the MCF-7 and 267B1 cells, both of which showed increased level of aerobic glycolysis with enhanced lactate production (Figs. 2C and 2D). In contrast, a substantial amount of mTOR proteins was detected in the mitochondrial fraction of MCF-10A cells and 267B1/Ki cells, though there was no difference in the total cellular level of mTOR. These results indicate that the localization of mTOR in mitochondrial is associated with oxidative phosphorylation (OXPHOS).

### Radiation-induced mTOR translocation: an adaptive switch of cellular bioenergetics

**Fig 3:**
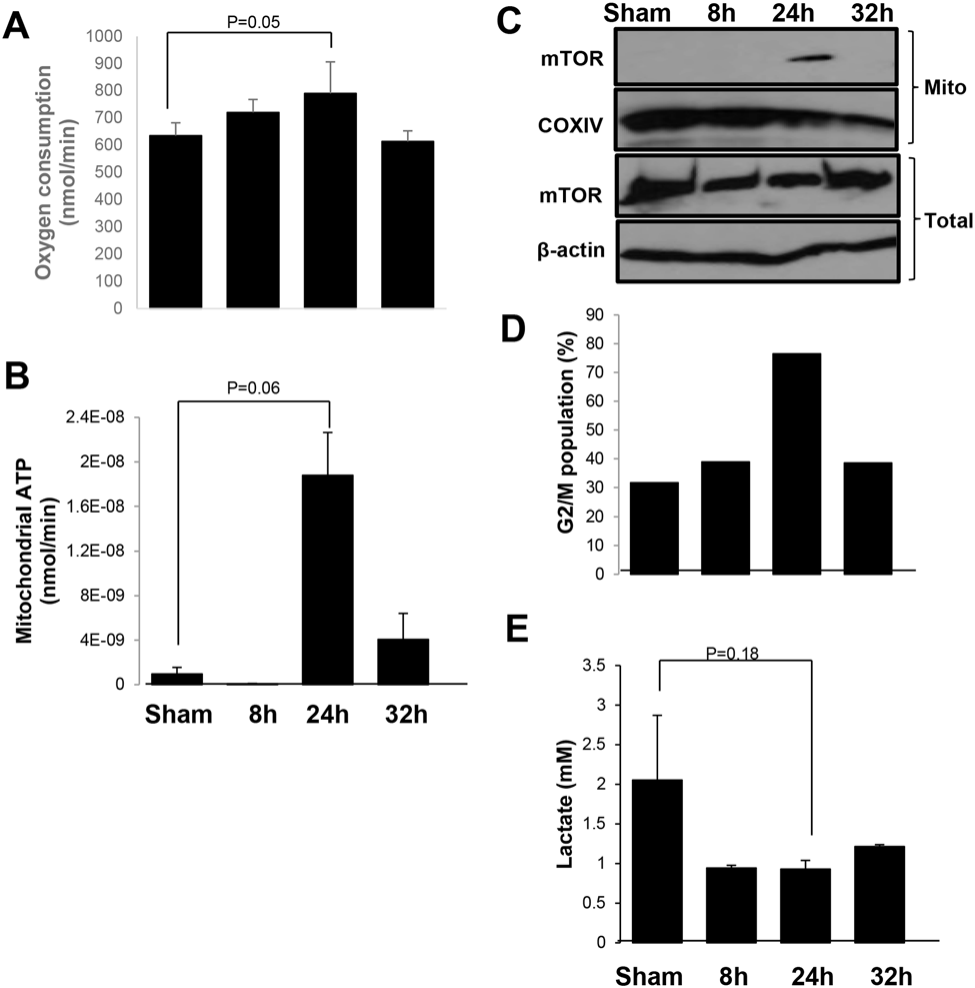
Radiation enhanced mitochondrial mTOR accumulation and switched bioenergetics from glycolysis to mitochondrial respiration. (A) Human breast cancer MCF-7 cells were treated with sham or radiation (5 Gy IR) and samples collected at the indicated time points to measure (A) oxygen consumption and (B) mitochondrial ATP production. (C) Western blotting of total cell lysate or mitochondrial fraction was performed to detect mTOR localization following sham or radiation. (D) Flow cytometry was performed to determine the percentage of G2/M arrest. (E) Lactate production in MCF-7 cells was measured by lactate assay kit. Data are mean ± SEM, p values were included in each data.

It is well established that mitochondria in cancer cells are dysfunctional and tumors predominantly use the aerobic glycolysis to produce cellular energy even in an environment with sufficient oxygen [1]. Recent studies, however, indicate that mitochondria in cancer cells still can be reactivated to increase respiratory activity [8]. In this study, we found that cellular bioenergetics can be switched from aerobic glycolysis to OXPHOS in MCF-7 cells irradiated with a single dose 5 Gy due to mTOR relocation to mitochondria. The mitochondrial mTOR relocation was matched with the time of the highest oxygen consumption and mitochondrial ATP production at 24 h post-irradiation (Figs. 3A and 3B). Consistent with the data shown in Fig. 1 that mitochondrial mTOR was linked with mitochondrial ATP production and oxygen consumption, the relocation of mTOR to mitochondria after radiation plays a role in mitochondrial bioenergetics that may be required for cellular damage repair and cell survival. To confirm that mitochondrial mTOR was critical for bioenergetics switch we irradiated MCF-7 cells and detected the expression level of mTOR at indicated time points. The total expression of mTOR showed no significant change after 5 Gy of radiation, but at 24 h post-irradiation mTOR relocated to mitochondria (Fig. 3C) which was also matched with the enhanced oxygen consumption and mitochondrial ATP generation with lowered lactate production. Flow cytometry was also performed using propidium iodide staining in MCF-7 cells to detect the percentage of G2/M arrest after 5 Gy (Figs. 3D and S1 Fig.). Radiation-induced DNA damage elicits a cell cycle arrest at G2/M phase [43]. The flow cytometry data showed that at 24 h post-5 Gy irradiation, most of cells were arrested in G2/M phase and lactate production was decreased, the lowest level at 24 h (Fig. 3E). Together, the data show that the translocation of mTOR to mitochondria enhances the switch of aerobic glycolysis to OXPHOS at the phase of G2/M arrest following genotoxic stress of ionizing radiation.

**Fig 4:**
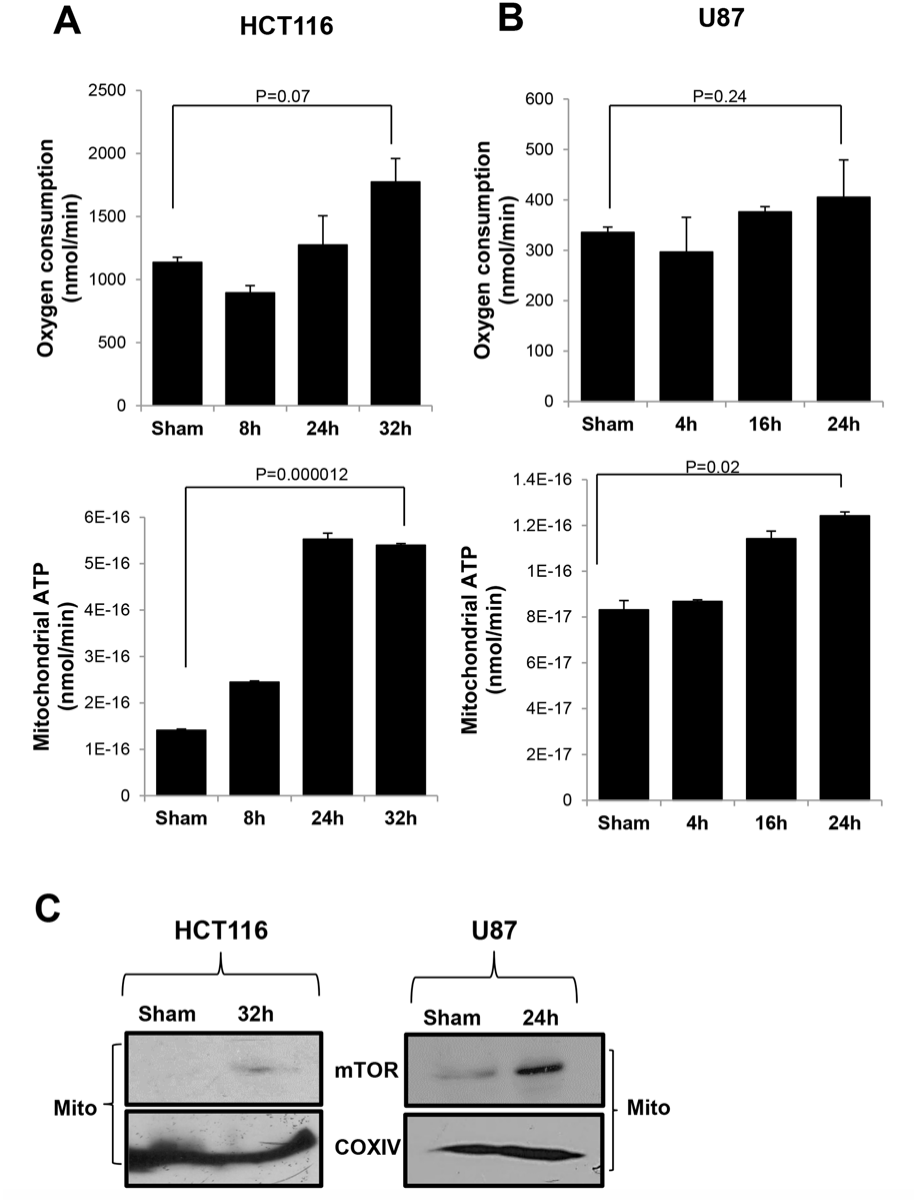
Radiation enhanced mitochondrial mTOR and mitochondrial respiration in other human cancer cells. (A) Human colon cancer HCT116 and (B) brain tumor U87 cells were irradiated or sham irradiated with IR and collected at indicated time points. Mitochondrial ATP production and oxygen consumption were measured to determine the enhancement of mitochondrial functions after radiation. (C) Western blotting of mitochondrial fractions of HCT116 and U87 were performed to detect mTOR localization at time points shown after radiation. Data are mean ± SEM, p-values were included in each data.

To confirm these findings in MCF7 cells, we repeated the experiment in three different tumor cell lines including human colon cancer HCT116 (Fig. 4A), brain tumor U87 (Fig. 4B) and murine breast cancer 4T1 (S2 Fig.). As seen in MCF-7 cells OXPHOS was enhanced at 32 h in HCT116 and 24 h in U87 post-irradiation, times when mTOR relocated to mitochondria in both cell types (Fig. 4C and S3 Fig). However, the murine breast cancer 4T1 cells showed both decreased mitochondrial ATP production, and no detectable mTOR translocation to mitochondria after radiation (S2A and S2B Figs.). Parallel results were obtained when 4T1 tumor cells were inoculated into mouse mammary glands and irradiated with 5 Gy of radiation (S4 Fig.). These results show that the relocation of mTOR to mitochondria after radiation is a key factor regulating mitochondrial functions under genotoxic stress condition and the enhancement of OXPHOS confirms that cancer cells still can use mitochondria to produce energy, particularly under conditions that replicate treatment.

### Inhibition of mTOR blocks radiation-induced bioenergetics switch

**Fig 5:**
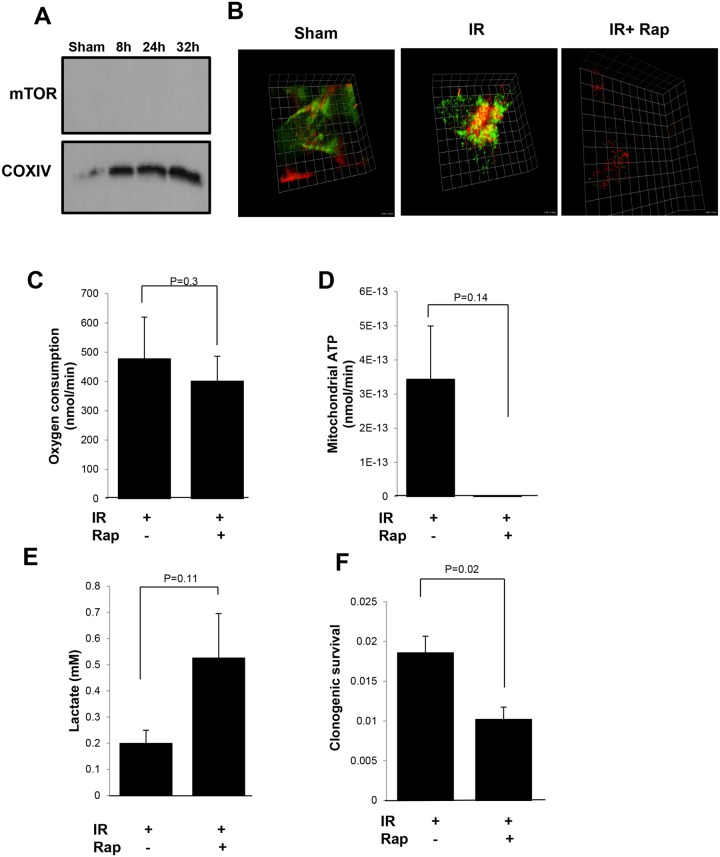
Rapamycin inhibited mitochondrial mTOR and blocked radiation-induced bioenergetics switching. (A) Western blot of mTOR with mitochondria isolated from MCF-7 cells treated with 20 ng/mL rapamycin for 30 min followed by IR. (B) 3D Images of co-localization of TOM40 (green), a mitochondrial outer membrane protein, and mTOR (red) in MCF-7 cells 24 h after treatment with sham, IR or radiation plus rapamycin (IR+Rap). (C) Mitochondrial oxygen consumption, (D) ATP generation, (E) lactate production and (F) clonogenic survival assay at 24 h post-IR in DMSO and rapamycin treated MCF-7 cells. Data are mean ± SEM, p-values were included in each data.

Next, we tested whether blocking mTOR could inhibit mTOR-mediated mitochondrial bioenergetics. Rapamycin, a well-characterized inhibitor of mTOR, binds to the FRB domain on mTOR which is believed to be the binding site for mTOR to mitochondria, via FKBP38 on the mitochondria outer surface [14]. We pre-treated MCF-7 cells with 20 ng/ml rapamycin for 30 minutes [44] to inhibit the translocation of mTOR to mitochondria, then irradiated cells with 5 Gy. After rapamycin treatment, the radiation-induced relocation of mTOR to mitochondria was totally blocked (Fig. 5A). Similar results were obtained when MCF-7 cells were stained with TOM40 (green), a mitochondria outer membrane protein, and mTOR (red). The images showed that the mTOR and TOM40 co-localized at 24 h after 5 Gy, and rapamycin treatment inhibited this co-localization (Fig. 5B and S5 Fig). In addition, we measured oxygen consumption, mitochondrial ATP production and lactate production at 24 h after-5 Gy, the peak of mitochondrial function after radiation, and found that cells with rapamycin-blocked mitochondrial mTOR, the bioenergetics cannot switch from aerobic glycolysis to OXPHOS after radiation (Figs. 5C, 5D and 5E). Potentially important in clinic, we found that rapamycin pre-treatment increased radiation lethality with about half clonogenicity remained in rapamycin treated cells measured by the clonogenic survival (Fig. 5F). These results indicate that mitochondrial mTOR functions as an enhancer for OXPHOS which is required for cellular response to genotoxic stress and survival. The relocation of mTOR to mitochondria is a critical factor that allows cells to reprogram cellular bioenergetics from aerobic glycolysis to OXPHOS to protect them from radiation injury.

### mTOR/Hexokinase II complex mediates the bioenergetics switch

**Fig 6:**
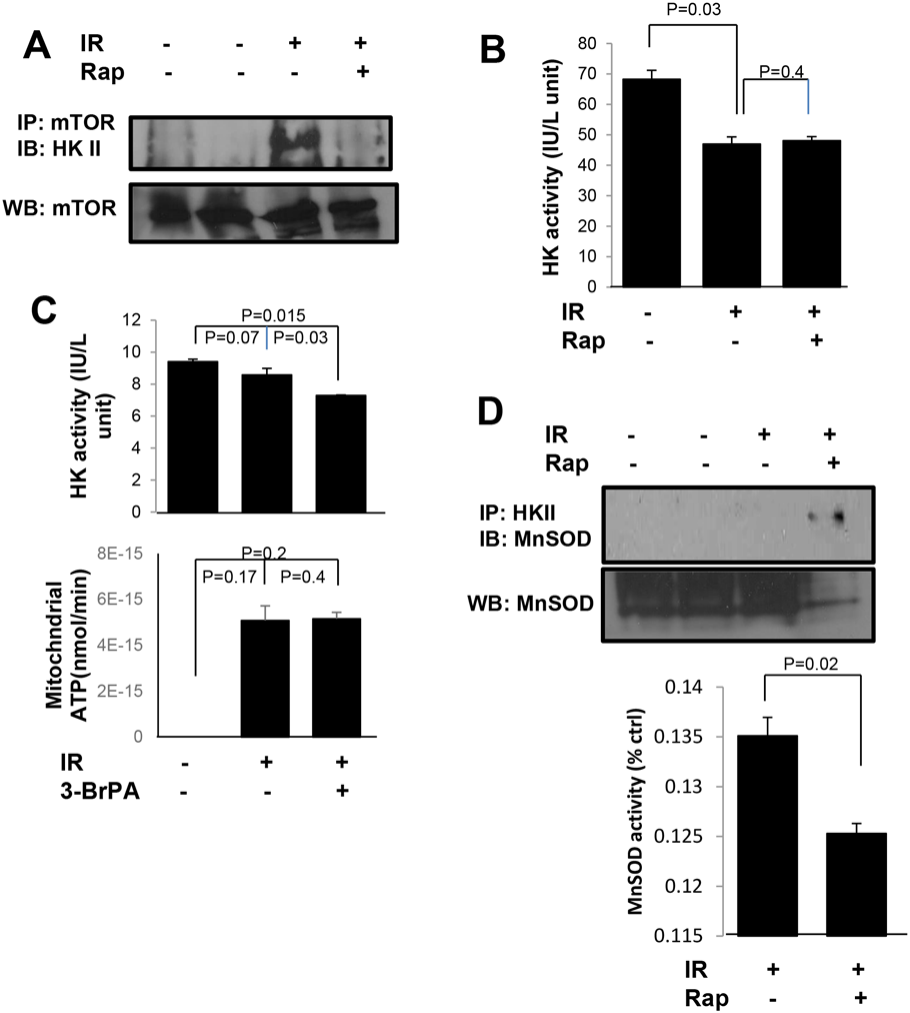
Mitochondrial adaptive bioenergetics response was blocked by inhibiting the interaction of mTOR/Hexokinase II [HK II]. (A) Co-immunoprecipitation of mTOR and HK II of MCF-7 cells treated with sham, IR with or without rapamycin. Cells were collected 24 h post-IR and IgG replaced anti-mTOR as a control. (B) HK II activity was measured in MCF-7 cells with the same treatment. (C) HK activity and mitochondrial ATP were measured in MCF-7 cells treated with sham, 24 h post-IR with DMSO and 24 h post-IR with the HK II inhibitor 3-BrPA. (D) Co-immunoprecipitation of HK II and MnSOD in MCF-7 cells treated with sham, 24 h post-IR, and 24 h post-IR with rapamycin pre-treatment. MnSOD activity was measured in MCF-7 cells at 24 h post-IR and 24 h post-IR with rapamycin. Data are mean ± SEM, p-values were included in each data.

To elucidate the mechanisms controlling the bioenergetics switch after radiation, we co-immunoprecipitated mTOR with another critical mitochondrial protein in glycolysis, Hexokinase II (HK II), to determine whether mTOR mediated mitochondrial bioenergetics is linked with HK II. HK II is in the first step of aerobic glycolysis; it is located on the outer membrane of mitochondria and phosphorylates glucose to glucose-6-phosphate. Therefore, it is highly possible that the interaction of mTOR and HK II can serve as a switcher on the mitochondrial surface to initiate the bioenergetics reprogramming. A recent study showed that mTORC1 and HK II formed a complex during glucose starvation in neonatal rat ventricular myocytes [45]. We hypothesized that radiation may trigger an interaction between mTOR and HK II to regulate bioenergetics. Co-immunoprecipitation (Co-IP) analysis in MCF-7 cells showed that mTOR did not interact with HK II in sham radiation control cells, but indeed, the mTOR and HK II complex was detected 24 h post-irradiation, the same time point where a switch in energy metabolism from aerobic glycolysis to OXPHOS was detected in MCF-7 cells (Fig. 6A). As expected, rapamycin inhibited the translocation of mTOR to mitochondria and prevented the interaction of mTOR and HK II.

This interaction, which is important for mitochondrial function enhancement, was also tested in mouse breast cancer 4T1 cells. Co-IP showed that the mTOR/HK II complex did not form at 24 h post-irradiation in 4T1 cells (S6 Fig.), a finding in accordance with the lack of a radiation-induced switch in bioenergetics for these cells. Thus, these result suggest that the mTOR/HK II interaction is required for promoting mitochondrial function in cancer cells and the regulation of mTOR on HK II is a key factor for the switch. To examine this possibility we measured Hexokinase activity to determine the regulation of mTOR on HK II after 5 Gy (Fig. 6B). We found that Hexokinase activity decreased at 24 h post-irradiation, when mTOR interacted with HK II, suggesting that mTOR-mediated inhibition of HK II may act as a biochemical switch of bioenergetics. To confirm that the inhibition of HK II controlled the switch, we treated MCF-7 cells with the HK II inhibitor, 3-BrPA (Fig. 6C). 3-BrPA can prevent the binding of HK II to mitochondria and inhibit the ability of HK II to phosphorylate glucose. We found that HK II activity was decreased at 24 h post-irradiation and further reduced by 3-BrPA treatment. The increase of mitochondrial ATP production was in accordance with the results obtained by rapamycin treatment in MCF-7 cells.

Our previous study showed that after low dose radiation, HK II can move into the mitochondrial matrix and interact with MnSOD [46]. We co-immunoprecipitated HK II and MnSOD in MCF-7 cells treated with rapamycin and/or radiation (Fig. 6D) and found that at 24 h post-irradiation, relocation of mTOR to mitochondria can prevent the influx of HK II into the mitochondrial matrix to form a complex with MnSOD. However, rapamycin treatment can rescue the HK II influx. HK II interaction with MnSOD results in reduced MnSOD activity (Fig. 6D) which may lead to higher ROS production and cell death. By blocking the interaction between HK II and MnSOD, mTOR enables the proper activation of MnSOD, which is required to scavenge elevated ROS resulting from enhanced OXPHOS. These results support the conclusion that the inhibitory function of mTOR on HK II can be induced by radiation and serves as a switch to turn mitochondrial functions on or off. Thus blocking this interaction may result in the inhibition of the acute adaptive response and cell survival under genotoxic stress condition caused by 5 Gy of radiation.

## Discussion

This report reveals a mechanism by which tumor cells are able to rapidly shift from aerobic glycolysis (Warburg Effect), to mitochondrial respiration after radiation exposure. As a result, cells will consume glucose more efficiently and generate more ATP following such genotoxic stressors by relocating mTOR to mitochondria. The mammalian target of rapamycin (mTOR) has been proven to be a central factor regulating cell proliferation, protein synthesis and cell survival [11]. In many cancer cells, mTOR is highly expressed [47–49] leading to the induction of several proteins that regulate aerobic glycolysis in cancer cells [24]. We compared two pairs of cell lines, MCF-10A and MCF-7, and 267B1 and 267B1/Ki, and found that the distribution of mitochondrial mTOR in cells was related to cellular metabolism status but not cell types. These results indicate that mitochondrial mTOR may not be a feature unique to tumor cells, but an opportunistic reprogramming of cellular metabolism from glycolysis to mitochondrial oxidative respiration, potentially providing extra energy to address genotoxic changes, enhancing tumor cell survival.

Therapeutic ionizing radiation can induce DNA damage and kill tumor cells, but tumors can still survive under this stress condition. Here, we found that radiation can induce the translocation of mTOR to mitochondria and induce changes in bioenergetics status following genotoxic stress. The mTOR-mediated metabolism switch in cancer cells provides a novel focus for the dynamic reprogramming of cellular bioenergetics. This may assist in the adaptation to changes in environmental conditions and, as our results show, may be linked to increased radiation resistance. In this context, pre-treatment of MCF-7 cells with the mTOR inhibitor, rapamycin, blocks the relocation of mTOR to mitochondria that we suggest inhibits the switch in bioenergetics after irradiation. Therefore, cells cannot switch metabolism to protect cells from the stress condition. The key role of mTOR in triggering the reprogramming of bioenergetics is seen in murine 4T1 cells, where the lack of a switch in bioenergetics status is associated with no mTOR accumulation on mitochondria. Therefore, not all tumors tested showed the ability of switching glycolysis to OXPHOS, supporting a key role for mitochondrial mTOR in “waking up” mitochondrial metabolic activity following genotoxic stress.

It has been well-established that reoxygenation of hypoxic regions occurs in irradiated tumors, probably due to the reduced oxygen demand from dead and dying cancer cells [9]. Reoxygenation of hypoxic regions in tumors is important in that a lack of molecular oxygen reduces the fixation of free radical mediated damage, increasing the resistance of cells to irradiation. Such damage fixation is a rapid event, of the order of microseconds, and thus is likely separate from any change in bioenergetics that will proceed much more slowly. Our recent studies also showed that radiation can induce mitochondrial protein influx (MPI), such as CDK 1, to enhance mitochondrial functions for adaptive response. The mitochondrial influx CDK1 can promote the activity of OXPHOS to induce anti-apoptotic response [35, 50, 51]. Although the replacement of mitochondrial respiration by aerobic glycolysis in tumor cells has been generally accepted, the partial activation of mitochondrial functions in tumors has only recently been reported [52], the overloading of the electron transport chain and increased mitochondrial superoxide activity may promote cancer metastasis [8, 53–55]. It is unknown whether tumor cells can utilize the increased oxygen for their cellular fuel supplement by converting glycolysis-mediated energy to the mitochondria-centered, oxygen-consuming ATP generation. Our study provide the first evidence indicating that a dynamic feature of reprogramming cellular bioenergetics is present in tumor cells, and a potential important factor that mediates such shifting in tumor bioenergetics from aerobic glycolysis to mitochondrial respiration is mTOR. Thus the mitochondria in tumor cells can be reactivated, or “awakened”, a feature we describe here as the “Warburg-Reversing Effect”, a process active during genotoxic stress, such as exposure to ionizing radiation. Radiation combined with rapamycin treatment has been studied before, where the combined treatment can enhance radiation sensitivity [56, 57]. Our results provide a mechanism for such sensitization whereby rapamycin treatment before radiation can prevent the relocation of mTOR to mitochondria. The relocation of mTOR is critical for the adaptive switch of bioenergetics in tumors enabling them to survive genotoxic stress.

**Fig 7:**
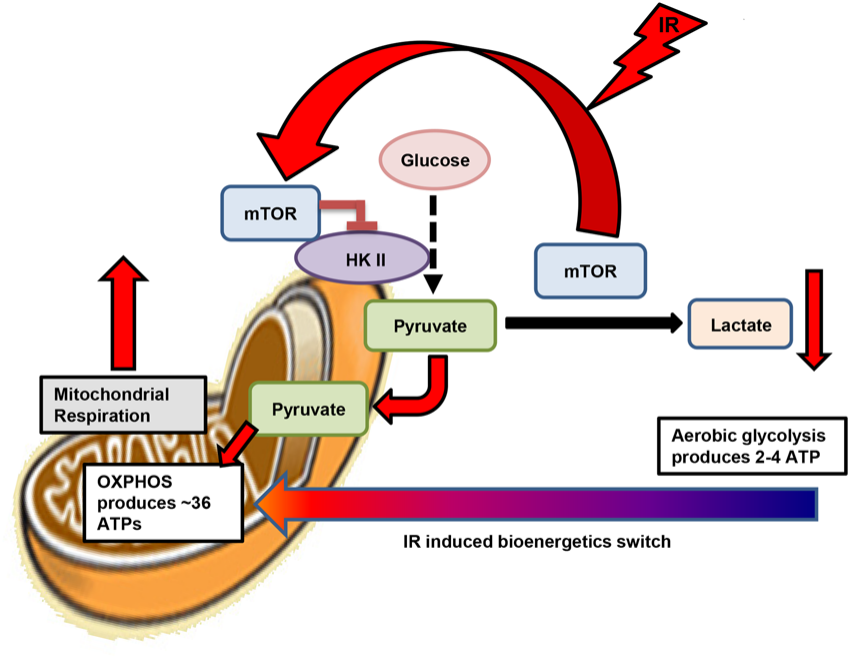
A proposed mechanism switching from glycolysis to mitochondrial oxidative phosphorylation by mTOR-mediated Hexokinase II [HK II] inhibition in tumor cells under genotoxic stress condition created by ionizing radiation (IR). In tumor cells, radiation induces the localization of mTOR from cytosol to mitochondrial surface where it interacts and inhibits the activity of Hexokinase II. The mitochondrial accumulated mTOR, as a result, will reduce aerobic glycolysis. Meanwhile, the mTOR-mediated HK II inhibition will reduce the HK II-mediated mitochondrial suppression, enhancing mitochondrial oxidative respiration. Such switch of aerobic glycolysis to oxidative phosphorylation in cancer cells under acute genotoxic stress conditions represents a stress-adaptive feature of tumor cells and may links to tumor resistance to anti-cancer therapy.

In order to examine the possible mechanism of rapamycin sensitization linked to mTOR, we focused on one of its possible targets, Hexokinase II (HK II). HK II is highly activated in cancer cells and is located on the outer membrane of mitochondria where it phosphorylates glucose in the first step of glycolysis [32]. It contains three (ST)Q motifs that can be phosphorylated by PIKK family members and mTOR belongs to this kinase family [58]. Therefore, mTOR may be able to phosphorylate HK II and regulate its activity. Study has shown that under starvation conditions, HK II can interact with mTORC1 to induce autophagy in neonatal rat ventricular myocytes. Therefore, the interaction of mTOR and HK II may be a factor to regulate cell survival under stress [45]. Our data showed that mTOR can directly interact with HK II to form a mTOR/HK II complex on mitochondria after 5 Gy. This interaction of mTOR and HK II can serve as a mitochondrial-based switch to convert energy metabolism from aerobic glycolysis to OXPHOS by inhibiting HK II activity. In support of this argument we showed that 3-BrPA, an HK II inhibitor, can prevent the binding of HK II to mitochondria and inhibit its activity in glycolysis. The inhibition of HK II after radiation can prevent HK II influx into mitochondrial matrix. In our studies we showed that after irradiation, mTOR relocates to mitochondrial outer membrane and binds to HK II, this interaction may dissociate HK II from mitochondria and inhibits its activity. The removal of HK II from mitochondria may turn on respiratory activity after radiation induced genotoxic stress condition and shift aerobic glycolysis to OXPHOS in order to produce sufficient ATP for DNA repair (Fig. 7). In Fig. 5F, our clonogenic survival experiment showed that rapamycin treated cells had a lower survival, potentially caused by the influx of HK II into the mitochondrial matrix. When rapamycin inhibits mTOR relocation to mitochondria, removing its interaction with HK II, HK II can then interact with MnSOD, inhibiting its function after irradiation, as seen in Fig. 6F. Thus the normal radical scavenging function of MnSOD is inhibited, increasing cell damage and thus cell death [51].

In summary, this work provides the evidence supporting a unique metabolic mechanism by which tumor cells can quickly shift their major energy metabolism from the aerobic glycolysis to mitochondrial respiration by relocating mTOR to mitochondria and inhibiting HK II activity. The inhibition of HK II function can slow down the aerobic glycolysis and increase OXPHOS activity which is required to increase tumor cell survival under genotoxic stress conditions. The dynamics of tumor cell bioenergetics under genotoxic stress conditions, especially the mTOR-mediated inhibition of glycolysis in tumor adaptive resistance needs to be further investigated.

## Supporting Information

S1 Fig Flow cytometry of MCF-7 after 5 Gy of radiation. Flow cytometry was performed to determine the percentage of G2/M arrest at irradiated sham, 8 h, 24 h and 32 h.
(TIF)

S2 Fig Bioenergetics of 4T1 cells after 5Gy of radiation. (A) Mitochondrial ATP production of 4T1 cells after 5 Gy of radiation at indicated time point. (B) mTOR western blotting of 4T1 mitochondrial fractions time course after 5 Gy of radiation.
(TIF)

S3 Fig Radiation induced mTOR relocation to mitochondria in U87 cell lines. U87 cell lines was treated with sham or radiation (5 Gy) and samples were collected at indicated time points for western blotting.
(TIF)

S4 Fig Bioenergetics of 4T1 xenograft tumor tissues after 5Gy of radiation. (A) Oxygen consumption and (B) mitochondrial ATP production were measured in two groups of mice at irradiated sham and 24 h post-irradiation. (C) mTOR western blotting of 4T1 xenograft tissues mitochondrial fractions of irradiated sham and 24 h post-irradiation was performed.
(TIF)

S5 Fig Image of TOM40 (green) and mTOR (red) co-localization after 5Gy of radiation. MCF-7 cells were irradiated under 5 Gy and collected at irradiated sham, 8h, 24h, 32 h and 24 h with rapamycin treatment. Cells were stained with TOM40 in green and mTOR in red.
(TIF)

S6 Fig No mTOR and HK II interaction after 5 Gy of radiation in 4T1 cells. Co-immunoprecipitation of mTOR and HK II in 4T1 cells with IgG control, irradiated sham, 24 h post-5 Gy irradiation and 24 h post-5 Gy irradiation with rapamycin treatment.
(TIF)

## References

[1] Vander Heiden MG, Cantley LC, Thompson CB. Understanding the Warburg effect: the metabolic requirements of cell proliferation. Science. 2009;324(5930):1029–33. 10.1126/science.1160809 19460998 PMC2849637

[2] Warburg O. On the origin of cancer cells. Scence. 1956;123(3191):309–70. 13298683 10.1126/science.123.3191.309

[3] Koppenol WH, Bounds PL, Dang CV. Otto Warburg's contributions to current concepts of cancer metabolism. Nat Rev Cancer. 2011;11(5):325–37. 10.1038/nrc3038 21508971

[4] Hu ZY, Xiao L, Bode AM, Dong Z, Cao Y. Glycolytic genes in cancer cells are more than glucose metabolic regulators. J Mol Med. 2014;92(8):837–45. 10.1007/s00109-014-1174-x 24906457

[5] Caro P, Kishan Amar U, Norberg E, Stanley IA, Chapuy B, Ficarro Scott B, et al Metabolic Signatures Uncover Distinct Targets in Molecular Subsets of Diffuse Large B Cell Lymphoma. Cancer Cell. 2012;22(4):547–60. 10.1016/j.ccr.2012.08.014 23079663 PMC3479446

[6] Pollak M. Targeting Oxidative Phosphorylation: Why, When, and How. Cancer Cell. 2013;23(3):263–4. 10.1016/j.ccr.2013.02.015 23518341

[7] Wang PY, Ma W, Park JY, Celi FS, Arena R, Choi JW, et al Increased oxidative metabolism in the Li-Fraumeni syndrome. New Eng J Med. 2013;368(11):1027–32. 10.1056/NEJMoa1214091 23484829 PMC4123210

[8] Martinez-Outschoorn UE, Pestell RG, Howell A, Tykocinski ML, Nagajyothi F, Machado FS, et al Energy transfer in "parasitic" cancer metabolism: mitochondria are the powerhouse and Achilles' heel of tumor cells. Cell Cycle. 2011;10(24):4208–16. 10.4161/cc.10.24.18487 22033146 PMC3272257

[9] Moeller BJ, Li YC, Dewhirst MW. Radiation activates HIF-1 to regulate vascular radiosensitivity in tumors: Role of reoxygenation, free radicals, and stress granules. Cancer Cell. 2004;5(5):429–41. 15144951 10.1016/s1535-6108(04)00115-1

[10] Easton JB, Houghton PJ. mTOR and cancer therapy. Oncogene. 2006;25(48):6436–46. 17041628 10.1038/sj.onc.1209886

[11] Yip CK, Murata K, Walz T, Sabatini DM, Kang SA. Structure of the human mTOR complex I and its implications for rapamycin inhibition. Mol Cell. 2010;38(5):768–74. 10.1016/j.molcel.2010.05.017 20542007 PMC2887672

[12] Ma XM, Blenis J. Molecular mechanisms of mTOR-mediated translational control. Nature reviews Mo Cell Biol. 2009;10[5]:307–18. 10.1038/nrm2672 19339977

[13] Chen H, Ma Z, Vanderwaal RP, Feng Z, Gonzalez-Suarez I, Wang S, et al The mTOR Inhibitor Rapamycin Suppresses DNA Double-Strand Break Repair. Radiat Res. 2011;175(2):214–24. 21268715 10.1667/rr2323.1 PMC4412148

[14] Ramanathan A, Schreiber SL. Direct control of mitochondrial function by mTOR. Proc Nat Acad Sci USA. 2009;106(52):22229–32. 10.1073/pnas.0912074106 20080789 PMC2796909

[15] Schieke SM, Phillips D, McCoy JP Jr, Aponte AM, Shen RF, Balaban RS, et al The mammalian target of rapamycin [mTOR] pathway regulates mitochondrial oxygen consumption and oxidative capacity. J Biol Chem. 2006;281(37):27643–52. 16847060 10.1074/jbc.M603536200

[16] Morita M, Gravel SP, Chenard V, Sikstrom K, Zheng L, Alain T, et al mTORC1 controls mitochondrial activity and biogenesis through 4E-BP-dependent translational regulation. Cell Met. 2013;18(5):698–711. 10.1016/j.cmet.2013.10.001 24206664

[17] Desai BN, Myers BR, Schreiber SL. FKBP12-rapamycin-associated protein associates with mitochondria and senses osmotic stress via mitochondrial dysfunction. Proc Nat Acad Sci USA. 2002;99(7):4319–24. 11930000 10.1073/pnas.261702698 PMC123646

[18] Paglin S, Lee NY, Nakar C, Fitzgerald M, Plotkin J, Deuel B, et al Rapamycin-sensitive pathway regulates mitochondrial membrane potential, autophagy, and survival in irradiated MCF-7 cells. Cancer Res. 2005;65(23):11061–70. 16322256 10.1158/0008-5472.CAN-05-1083

[19] Cunningham JT, Rodgers JT, Arlow DH, Vazquez F, Mootha VK, Puigserver P. mTOR controls mitochondrial oxidative function through a YY1-PGC-1alpha transcriptional complex. Nature. 2007;450(7170):736–40. 18046414 10.1038/nature06322

[20] Akhavan D, Cloughesy TF, Mischel PS. mTOR signaling in glioblastoma: lessons learned from bench to bedside. Neuro-oncology. 2010;12(8):882–9. 10.1093/neuonc/noq052 20472883 PMC2940679

[21] Dunlop EA, Tee AR. Mammalian target of rapamycin complex 1: signalling inputs, substrates and feedback mechanisms. Cell Sign. 2009;21(6):827–35. 10.1016/j.cellsig.2009.01.012 19166929

[22] Laplante M, Sabatini DM. mTOR signaling at a glance. J Cell Sci. 2009;122 (Pt20):3589–94. 10.1242/jcs.051011 19812304 PMC2758797

[23] Sengupta S, Peterson TR, Sabatini DM. Regulation of the mTOR complex 1 pathway by nutrients, growth factors, and stress. Mol Cell. 2010;40(2):310–22. 10.1016/j.molcel.2010.09.026 20965424 PMC2993060

[24] Laplante M, Sabatini DM. mTOR signaling in growth control and disease. Cell. 2012;149(2):274–93. 10.1016/j.cell.2012.03.017 22500797 PMC3331679

[25] Masui K, Tanaka K, Akhavan D, Babic I, Gini B, Matsutani T, et al mTOR complex 2 controls glycolytic metabolism in glioblastoma through FoxO acetylation and upregulation of c-Myc. Cell metabolism. 2013;18(50:726–39. 24140020 10.1016/j.cmet.2013.09.013 PMC3840163

[26] Crowder RJ, Ellis MJ. Treating breast cancer through novel inhibitors of the phosphatidylinositol 3'-kinase pathway. Breast cancer research: BCR. 2005;7(5):212–4. 16168140 10.1186/bcr1307 PMC1242159

[27] Cho DC, Cohen MB, Panka DJ, Collins M, Ghebremichael M, Atkins MB, et al The efficacy of the novel dual PI3-kinase/mTOR inhibitor NVP-BEZ235 compared with rapamycin in renal cell carcinoma. Clin Cancer Res. 2010;16(14):3628–38. 10.1158/1078-0432.CCR-09-3022 20606035 PMC2905505

[28] Serra V, Markman B, Scaltriti M, Eichhorn PJA, Valero V, Guzman M, et al NVP-BEZ235, a Dual PI3K/mTOR Inhibitor, Prevents PI3K Signaling and Inhibits the Growth of Cancer Cells with Activating PI3K Mutations. Cancer Res. 2008;68(19):8022–30. 10.1158/0008-5472.CAN-08-1385 18829560

[29] Cao P, Maira SM, García-Echeverría C, Hedley DW. Activity of a novel, dual PI3-kinase/mTor inhibitor NVP-BEZ235 against primary human pancreatic cancers grown as orthotopic xenografts. Brit J Cancer. 2009;100(8):1267–76. 10.1038/sj.bjc.6604995 19319133 PMC2676548

[30] Sun Q, Chen X, Ma J, Peng H, Wang F, Zha X, et al Mammalian target of rapamycin up-regulation of pyruvate kinase isoenzyme type M2 is critical for aerobic glycolysis and tumor growth. Proc Nat Acad Sci USA. 2011;108(10):4129–34. 10.1073/pnas.1014769108 21325052 PMC3054028

[31] Wittwer JA, Robbins D, Wang F, Codarin S, Shen X, Kevil CG, et al Enhancing mitochondrial respiration suppresses tumor promoter TPA-induced PKM2 expression and cell transformation in skin epidermal JB6 cells. Cancer prevention research. 2011;4(9):1476–84. 10.1158/1940-6207.CAPR-11-0028 21673231 PMC4827450

[32] Mathupala SP, Ko YH, Pedersen PL. Hexokinase II: cancer's double-edged sword acting as both facilitator and gatekeeper of malignancy when bound to mitochondria. Oncogene. 2006;25(34):4777–86. 16892090 10.1038/sj.onc.1209603 PMC3385868

[33] Fang R, Xiao T, Fang Z, Sun Y, Li F, Gao Y, et al MicroRNA-143 [miR-143] regulates cancer glycolysis via targeting hexokinase 2 gene. J Biol Chem. 2012;287(27):23227–35. 10.1074/jbc.M112.373084 22593586 PMC3391126

[34] Fujikawa M, Yoshida M. A sensitive, simple assay of mitochondrial ATP synthesis of cultured mammalian cells suitable for high-throughput analysis. Bioch Biophys Res Com. 2010;401(40:538–43. 10.1016/j.bbrc.2010.09.089 20875793

[35] Wang MFZ, Candas D, Zhang TQ, Qin L, Eldridge A, Wachsmann-Hogiu S, et al Cyclin B1/Cdk1 Coordinates Mitochondrial Respiration for Cell-Cycle G2/M Progression. Dev Cell. 2014;29(2):217–32. 10.1016/j.devcel.2014.03.012 24746669 PMC4156313

[36] Wang T, Hu YC, Dong S, Fan M, Tamae D, Ozeki M, et al Co-activation of ERK, NF-kappaB, and GADD45beta in response to ionizing radiation. J Biol Chem. 2005;280 (13):12593–601. 15642734 10.1074/jbc.M410982200 PMC4130153

[37] Kilkenny WJBC, Cuthill IC, Emerson M, Altman DG. Improving Bioscience Research Reporting: The ARRIVE Guidelines for Reporting Animal Research. PLoS Biol. 2010;8(6):1–5. 10.1371/journal.pbio.1000412 PMC2893951 20613859

[38] Ahmed KM, Fan M, Nantajit D, Cao N, Li JJ. Cyclin D1 in low-dose radiation-induced adaptive resistance. Oncogene. 2008;27(53):6738–48. 10.1038/onc.2008.265 18695676 PMC6759063

[39] Khacho M, Tarabay M, Patten D, Khacho P, MacLaurin JG, Guadagno J, et al Acidosis overrides oxygen deprivation to maintain mitochondrial function and cell survival. Nature Commun. 2014;5:3550 10.1038/ncomms4550 24686499 PMC3988820

[40] Leeuwenburgh BDaC. Method for measuring ATP production in isolated mitochondria: ATP production in brain and liver mitochondria of Fischer-344 rats with age and caloric restriction. Am J Physiol Regul Integr Comp Physiol. 2003;285:R1259–R67,. 12855419 10.1152/ajpregu.00264.2003

[41] Chen LY, Zhang Y, Zhang Q, Li H, Luo Z, Fang H, et al Mitochondrial localization of telomeric protein TIN2 links telomere regulation to metabolic control. Mol Cell. 2012;47(6):839–50. 10.1016/j.molcel.2012.07.002 22885005 PMC3462252

[42] Oruganty-Das A, Ng T, Udagawa T, Goh EL, Richter JD. Translational control of mitochondrial energy production mediates neuron morphogenesis. Cell Met. 2012;16(6):789–800. 10.1016/j.cmet.2012.11.002 PMC3597101 23217258

[43] Chapman JR, Taylor MR, Boulton SJ. Playing the end game: DNA double-strand break repair pathway choice. Mol Cell. 2012;47(4):497–510. 10.1016/j.molcel.2012.07.029 22920291

[44] Holz MK, Blenis J. Identification of S6 kinase 1 as a novel mammalian target of rapamycin [mTOR]-phosphorylating kinase. J Biol Chem. 2005;280(28):26089–93. 15905173 10.1074/jbc.M504045200

[45] Roberts DJ, Tan-Sah VP, Ding EY, Smith JM, Miyamoto S. Hexokinase-II positively regulates glucose starvation-induced autophagy through TORC1 inhibition. Mol Cell. 2014;53(4):521–33. 10.1016/j.molcel.2013.12.019 24462113 PMC3943874

[46] Eldridge A, Fan M, Woloschak G, Grdina DJ, Chromy BA, Li JJ. Manganese superoxide dismutase interacts with a large scale of cellular and mitochondrial proteins in low-dose radiation-induced adaptive radioprotection. Free Radic Biol Med. 2012;53(10):1838–47. 10.1016/j.freeradbiomed.2012.08.589 23000060 PMC3494792

[47] Beauchamp EM, Platanias LC. The evolution of the TOR pathway and its role in cancer. Oncogene. 2013;32(34):3923–32. 10.1038/onc.2012.567 23246968

[48] Sabatini DM. mTOR and cancer: insights into a complex relationship. Nat Rev Cancer. 2006;6:729–34. 16915295 10.1038/nrc1974

[49] Zoncu R, Efeyan A, Sabatini DM. mTOR: from growth signal integration to cancer, diabetes and ageing. Nat Rev Mol Cell Biol. 2011;12(1):21–35. 10.1038/nrm3025 21157483 PMC3390257

[50] Blagosklonny MV, Nantajit D, Fan M, Duru N, Wen Y, Reed JC, et al Cyclin B1/Cdk1 Phosphorylation of Mitochondrial p53 Induces Anti-Apoptotic Response. PloS One. 2010;5(8):e12341 10.1371/journal.pone.0012341 20808790 PMC2925892

[51] Candas D, Li JJ. MnSOD in Oxidative Stress Response-Potential RegulationviaMitochondrial Protein Influx. Antiox & Redox Sign. 2014;20(10):1599–617. 10.1071/RD14474 23581847 PMC3942709

[52] Bartoletti-Stella A, Mariani E, Kurelac I, Maresca A, Caratozzolo MF, Iommarini L, et al Gamma rays induce a p53-independent mitochondrial biogenesis that is counter-regulated by HIF1alpha. Cell Death & Dis. 2013;4:e663. 10.1038/cddis.2013.187 PMC3702280 23764844

[53] Porporato PE, Payen VL, Perez-Escuredo J, De Saedeleer CJ, Danhier P, Copetti T, et al A mitochondrial switch promotes tumor metastasis. Cell Rep. 2014;8(3):754–66. 10.1016/j.celrep.2014.06.043 25066121

[54] Kim JH, Jenrow KA, Brown SL. Mechanisms of radiation-induced normal tissue toxicity and implications for future clinical trials. Rad Onc J. 2014;32(3):103–15. 10.3857/roj.2014.32.3.103 PMC4194292 25324981

[55] Tan AS, Baty JW, Dong LF, Bezawork-Geleta A, Endaya B, Goodwin J, et al Mitochondrial Genome Acquisition Restores Respiratory Function and Tumorigenic Potential of Cancer Cells without Mitochondrial DNA. Cell Met. 2015;21(1):81–94. 10.1016/j.cmet.2014.12.003 25565207

[56] Chang L, Graham PH, Hao J, Ni J, Bucci J, Cozzi PJ, et al PI3K/Akt/mTOR pathway inhibitors enhance radiosensitivity in radioresistant prostate cancer cells through inducing apoptosis, reducing autophagy, suppressing NHEJ and HR repair pathways. Cell Death & Dis. 2014;5:e1437. 10.1038/cddis.2014.415 PMC4237243 25275598

[57] Kim KW CJM, Jung DK, Lu B. NVP-BEZ-235 enhances radiosensitization via blockade of the PI3K/mTOR pathway in cisplatin-resistant non-small cell lung carcinoma. Genes Cancer. 2014;5:293–302. 25221647 10.18632/genesandcancer.27 PMC4162139

[58] Yang H, Rudge DG, Koos JD, Vaidialingam B, Yang HJ, Pavletich NP. mTOR kinase structure, mechanism and regulation. Nature. 2013;497(7448):217–23. 10.1038/nature12122 23636326 PMC4512754

