# Supplementary figures and images for: Tumor Cells Switch to Mitochondrial Oxidative Phosphorylation under Radiation via mTOR-Mediated Hexokinase II Inhibition - A Warburg-Reversing Effect
Source: PLoS One. 2015 Mar 25;10(3):e0121046. doi: 10.1371/journal.pone.0121046 (PMC4373728; doi:10.1371/journal.pone.0121046)

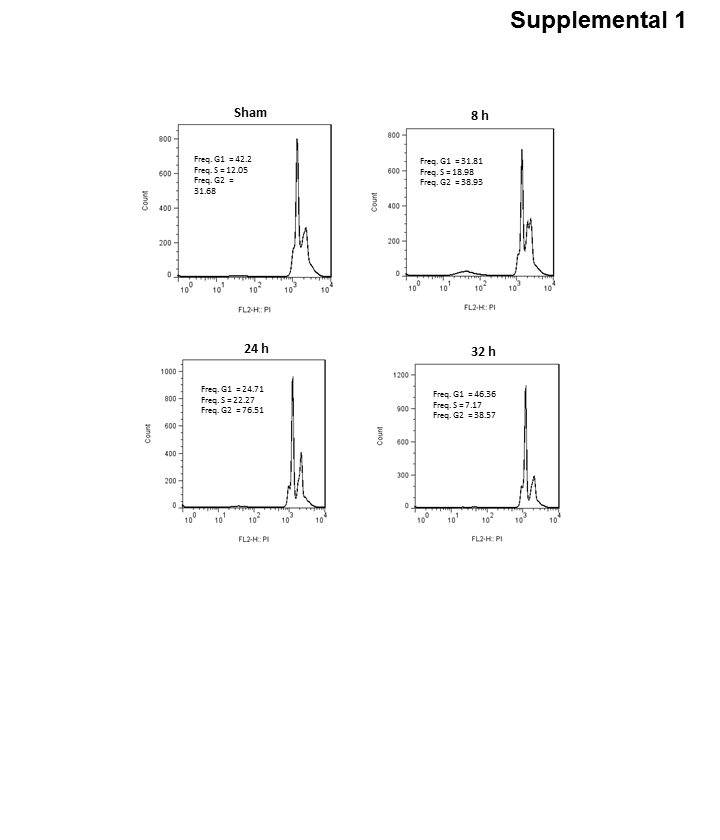

Supplement: S1 Fig — Flow cytometry was performed to determine the percentage of G2/M arrest at irradiated sham, 8 h, 24 h and 32 h. (TIF) [file pone.0121046.s001.tif]

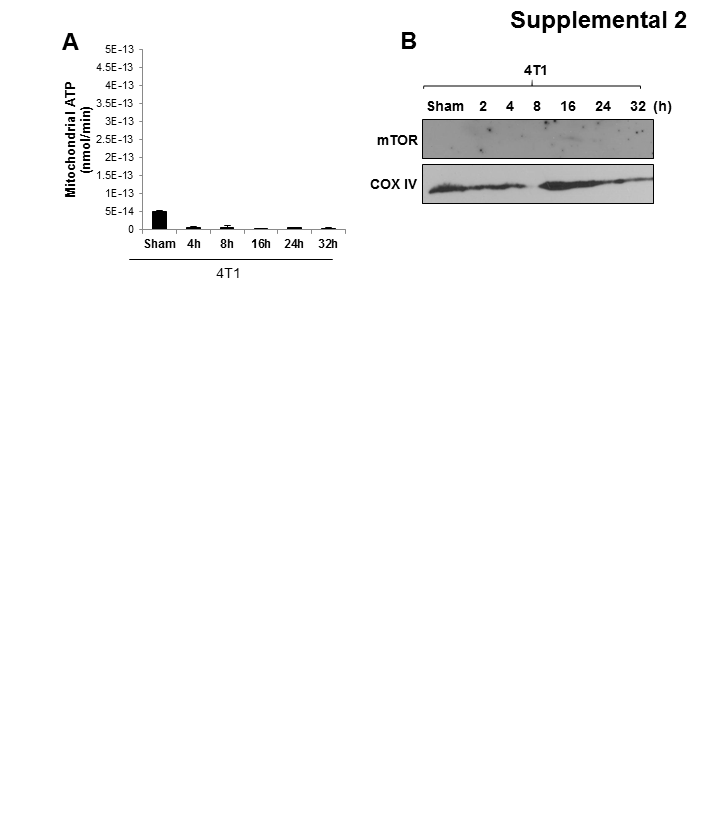

Supplement: S2 Fig — (A) Mitochondrial ATP production of 4T1 cells after 5 Gy of radiation at indicated time point. (B) mTOR western blotting of 4T1 mitochondrial fractions time course after 5 Gy of radiation. (TIF) [file pone.0121046.s002.tif]

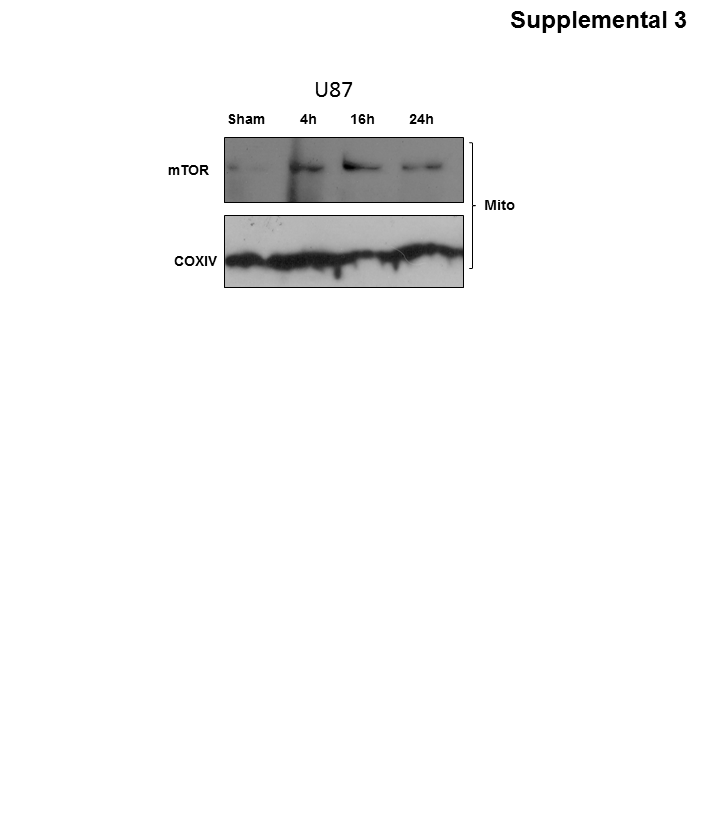

Supplement: S3 Fig — U87 cell lines was treated with sham or radiation (5 Gy) and samples were collected at indicated time points for western blotting. (TIF) [file pone.0121046.s003.tif]

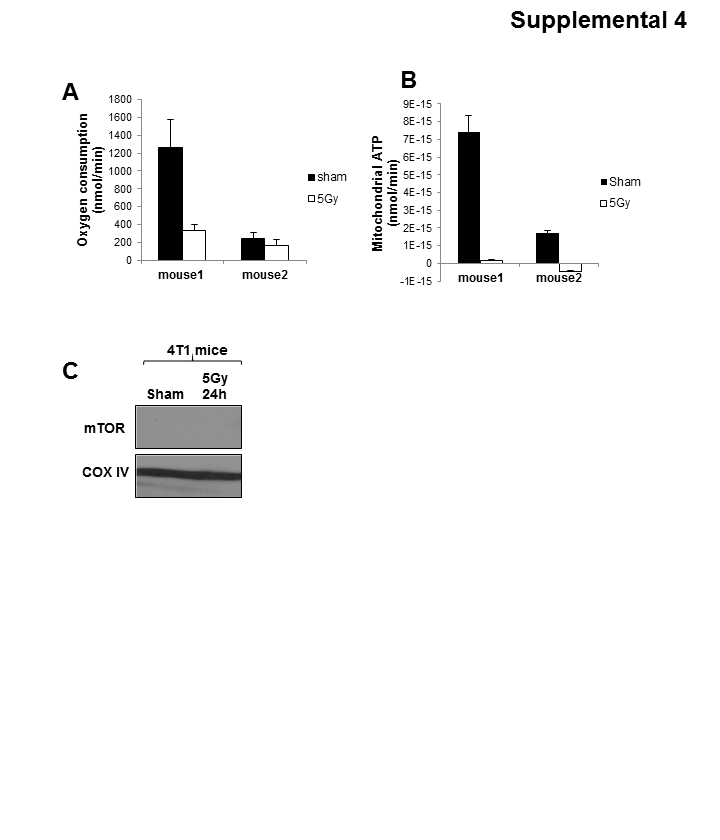

Supplement: S4 Fig — (A) Oxygen consumption and (B) mitochondrial ATP production were measured in two groups of mice at irradiated sham and 24 h post-irradiation. (C) mTOR western blotting of 4T1 xenograft tissues mitochondrial fractions of irradiated sham and 24 h post-irradiation was performed. (TIF) [file pone.0121046.s004.tif]

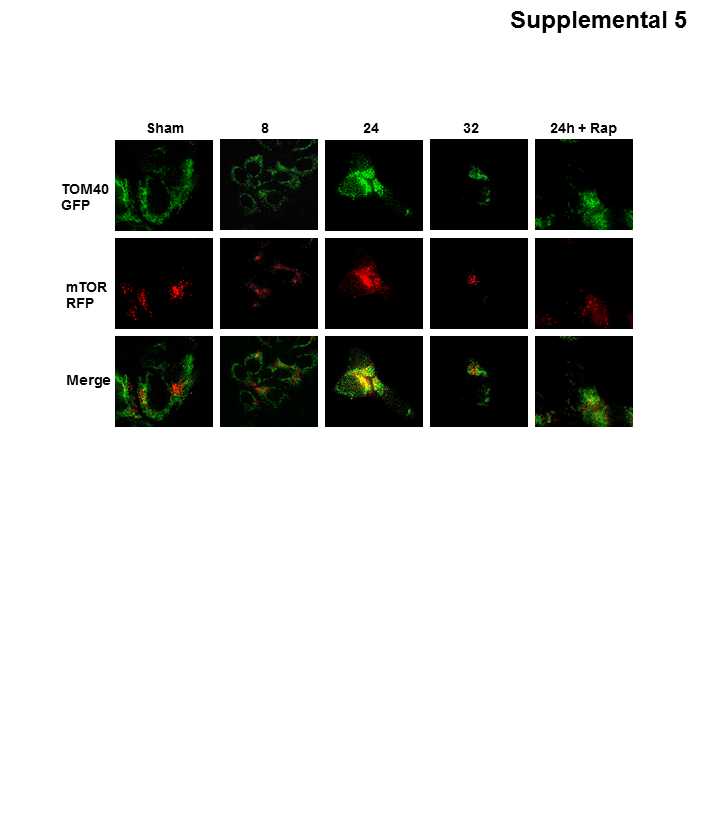

Supplement: S5 Fig — MCF-7 cells were irradiated under 5 Gy and collected at irradiated sham, 8h, 24h, 32 h and 24 h with rapamycin treatment. Cells were stained with TOM40 in green and mTOR in red. (TIF) [file pone.0121046.s005.tif]

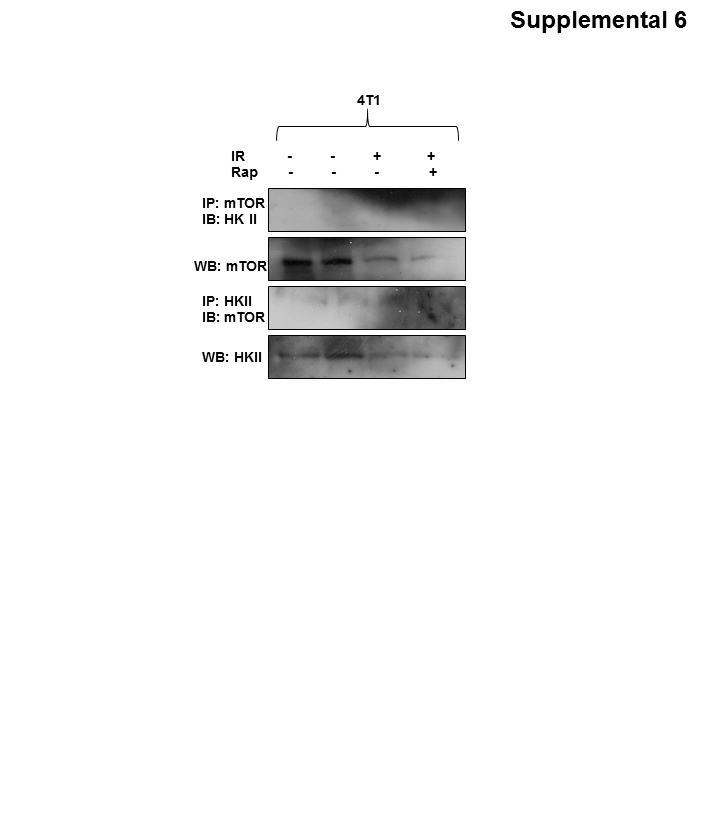

Supplement: S6 Fig — Co-immunoprecipitation of mTOR and HK II in 4T1 cells with IgG control, irradiated sham, 24 h post-5 Gy irradiation and 24 h post-5 Gy irradiation with rapamycin treatment. (TIF) [file pone.0121046.s006.tif]
